# Thermochromic and Femtosecond-Laser-Induced Damage Performance of Tungsten-Doped Vanadium Dioxide Films Prepared Using an Alloy Target

**DOI:** 10.3390/ma11091724

**Published:** 2018-09-14

**Authors:** Mao-Dong Zhu, Chong Shan, Cheng Li, Hu Wang, Hong-Ji Qi, Dong-Ping Zhang, Wei Zhong Lv

**Affiliations:** 1 Key Laboratory of Materials for High Power Laser, Shanghai Institute of Optics and Fine Mechanics, Chinese Academy of Sciences, Shanghai 201800, China; zhumaodong@siom.ac.cn (M.-D.Z.); licheng@siom.ac.cn (C.L.); wanghu@siom.ac.cn (H.W.); 2 University of Chinese Academy of Sciences, Beijing 100049, China; 3 Shanghai Institute of Laser Plasma, China Academy of Engineering Physics, Shanghai 201800, China; Sichongshan@126.com; 4 Shenzhen Key Laboratory of Advanced Thin Film and Applications, College of Physics and Energy, Shenzhen University, Shenzhen 518060, China; 5 College of Chemistry and Environmental Engineering, Shenzhen University, Shenzhen 518060, China; lvwzh@szu.edu.cn

**Keywords:** vanadium dioxide, metal doping, laser-induced damage, thermochromic performance

## Abstract

Thermochromic tungsten-doped VO_2_ thin films were successfully fabricated using a W-V alloy target. X-ray diffraction analyses showed that the W-doped VO_2_ film had a preferred orientation of (011), and that the doping did not degrade the film crystallinity compared with that of the pure film. X-ray photoelectron spectroscopy and energy-dispersive spectroscopy showed that the doped 0.81 atom% tungsten replaced vanadium in the lattice of the film. The metal–insulator transition temperature of the W-doped VO_2_ film was reduced to 35.5 °C, which is close to room temperature. Additionally, the infrared transmittance modulation of the W-doped film at λ = 2500 nm reached 56%, indicating an excellent switching efficiency. The damage behavior of the W-doped VO_2_ film under a femtosecond-laser irradiation was experimentally investigated. Our results revealed that defect-related damages induced by the femtosecond laser are relevant for W-doped VO_2_ films. This study provides valuable insights into VO_2_ films for potential applications in laser protection.

## 1. Introduction

With the development of the photoelectric detection technology, the effects of high-power lasers on photoelectric detectors have been extensively studied, including high-power-laser-induced damage mechanisms and damage threshold of photoelectric detectors. Protection against high-power lasers using suitable materials has also been studied. Advanced laser protection materials are usually based on conventional linear optics [1]. However, such protection systems not only absorb but also reflect waves of the same wavelength. In other words, the system protects the photoelectric detector from the strong laser; however, it also obstructs the weak waves that carry signals [2]. Therefore, they do not satisfy the requirements for the development of high-power-laser protection systems.

VO_2_ is a promising material for development of laser protection systems owing to its thermochromic phase-transition characteristics, low threshold laser energy, and fast response [3]. In general, VO_2_ is a typical thermochromic material which undergoes an extremely abrupt first-order phase transformation from a monoclinic structure to a rutile structure at approximately 68 °C, accompanied by significant changes in its electrical conductivity and infrared (IR) optical transmittance [4,5]. In its low-temperature states (below the transition temperature), VO_2_ exhibits semiconductor properties, such as a high IR transmission, while in its high-temperature states (above the transition temperature), it exhibits metallic properties, such as a low IR transmission [6]. The light-induced ultrafast phase transition of VO_2_ occurs in ~10^−13^ s [7]. These advantages make VO_2_ promising for various applications including optical switches [8] and solar heat management [9].

When a high-power laser irradiates a photoelectric detector protected by VO_2_ films, the laser initially interacts with the VO_2_ films, inducing thermochromic phase transitions. The VO_2_ films complete the phase transition process before the laser damages the detector; the films then behave as in a metallic state reflecting a large amount of IR energy to reduce the damaging laser energy. This may be an effective approach for high-power-laser protection. The extremely fast response time is crucial for VO_2_ films used in the field of laser protection. Cavalleri et al. had studied the phase-transition dynamics of VO_2_ at an extremely small time-scale upon laser excitation using optical pump-probe techniques [10].

The high phase-transition temperature of VO_2_ of 68 °C limits its applications. Modulation of the transition temperature to room temperature is indispensable for many practical applications, including smart windows. For laser-protection applications, a VO_2_ film with a high transition temperature could provide a high laser energy input threshold and large response time. The transmitted laser energy may exceed the damage threshold of the detector before the transition of the film; in such a case, the VO_2_ film has a low protection efficiency. The laser energy that should be absorbed by the VO_2_ films for phase-transition completion can be obtained by [1]:

(1) $$E=C\;\left(T_{p}-T_{a}\right)$$

where *C* is the heat capacity of the VO_2_ thin film, while *T_p_* and *T_a_* are the phase-transition temperature of the VO_2_ film and environment temperature, respectively. With the decrease in *T_p_ − T_a_*, the response time of VO_2_ decreases.

Therefore, the phase-transition temperature of the VO_2_ film should be reduced to room temperature. In general, doping with high-valence cations, such as Mo^6+^, Ta^5+^, Nb^5+^, and W^6+^, into the VO_2_ lattice may be a useful approach to reduce the transition temperature [11,12,13]. Among these cations, W^6+^ could be the most effective doping cation to regulate the phase-transition temperature of the VO_2_ film.

Various methods, such as the sol-gel process [14], chemical vapor deposition (CVD) [15], hydrothermal growth [16], and magnetron sputtering [17], have been employed for preparation of tungsten-doped VO_2_ films with specific thermochromic performances. Among them, the magnetron sputtering technology has advantages for applications in smart windows and laser-protection systems owing to its high packing density [18]. Some studies on tungsten-doped VO_2_ films prepared by the co-sputtering technique have been reported [5,19]. However, using this method, it could be difficult to precisely control the concentration of the doped tungsten and uniformity of the deposited films. In this study, we used a vanadium–tungsten alloy target (atomic percentage of W: 0.81%) to deposit tungsten-doped VO_2_ films with a uniform concentration of the doped tungsten. The phase-transition temperature of the W-doped VO_2_ film can reach a low value of 35.5 °C, which is near room temperature.

The laser-damage behavior of VO_2_ films, significant for studies of laser-protection systems, has not been extensively investigated. A femtosecond-(fs)-laser-induced breakdown of a film is strongly related with the intrinsic properties of the material. Therefore, in this study, we focused on the fs-laser-induced damage mechanism and laser damage threshold of W-doped VO_2_ films.

## 2. Experimental Methods

**Figure 1:**
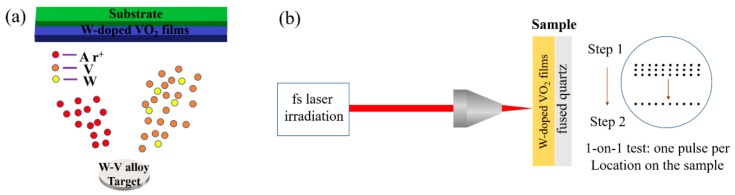
(**a**) Schematic of the DC magnetron sputtering system and vanadium-tungsten alloy target. (**b**) Schematic of the setup for the fs-laser damage tests.

W-doped VO_2_ thin films were deposited on fused quartz through DC reactive magnetron sputtering. Regarding the reduction in the phase-transition temperature to room temperature, it has been reported [20,21,22] that the tungsten doping linearly decreases the transition temperature of a VO_2_ film at an approximate rate of 23 °C/atom%. Based on these studies, a vanadium–tungsten alloy target (atomic percentage of W: 0.81%) was used to prepare the films. The diameter of the target was 60 mm. The distance between the target and glass substrate was approximately 10 cm. Before deposition, the vacuum chamber was pumped to a pressure of approximately 6 × 10^−4^ Pa. A pre-sputtering of the vanadium–tungsten alloy target was carried out for approximately 10 min. Ar (99.99%) and O_2_ (99.99%) were used as the sputtering and reactive gases, respectively. For the deposition of the W-doped films, O_2_ was mixed with Ar; the flow rates of Ar and O_2_ were 40 sccm and 1.8 sccm, respectively. The substrate temperature was maintained at 320 °C. The fused quartz substrate was put on the surface of the conductive fixture, which was connected to a DC source with a bias voltage supply of −125 V during the deposition. The sputtering power of the vanadium–tungsten alloy target was 80 W. A schematic of the DC magnetron sputtering process and vanadium–tungsten alloy target is shown in Figure 1a. A schematic of the setup for the fs-laser damage test is shown in Figure 1b. Laser-induced damage threshold (LIDT) tests were performed in the one-on-one mode.

**Figure 2:**
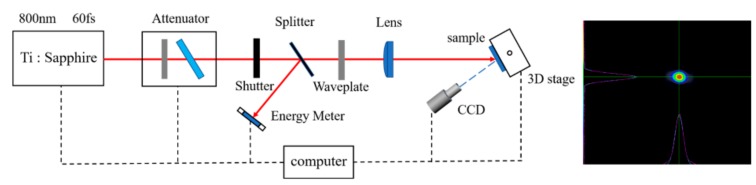
(**Left**) Experimental setup for laser damage tests at 60 fs and 800 nm. (**Right**) Image of the laser beam intensity distribution at the sample location.

The configuration for the damage tests is illustrated in Figure 2. Laser pulses were generated using a Ti: sapphire regenerative amplifier laser system (Spectra physics), which produced linearly polarized laser pulses with a duration of 60 fs and wavelength of 800 nm at a frequency of 10 Hz. As shown in Figure 2, a *λ*/2-plate and polarizing beam splitter were used as an attenuator to change the energy of the pulses. A shutter was used to extract a single pulse. Another *λ*/2-plate in the bottom part was used to change the polarization of the pulses. The samples were mounted on a motorized *x*–*z* rotation stage and positioned at the focal plane of the lens; the *e*^−2^-intensity diameter of the laser spot at the focus was measured to be approximately 320 μm. The pulse energy was measured in the reference path, split using a beam splitter. In-situ imaging of the front surface of the sample combined with ex-situ optical microscopy observations was carried out to monitor the occurrence of damage.

X-ray diffraction (XRD) (Ultima IV, Rigaku, Tokyo, Japan) analyses were carried out to evaluate the crystalline structures of the W-doped films. The chemical composition of the W-doped VO_2_ film was evaluated using a X-ray photoelectron spectroscope (XPS) (Thermo K-Alpha XPS, Thermo Scientific, Waltham, MA, USA). A four-point probe system was used to characterize the temperature-dependent sheet resistances of the W-doped VO_2_ films. During the measurement, the film temperature was controlled in the range of room temperature to 70 °C. The normal-incidence spectral transmittances of the W-doped films at temperatures below and above the transition temperatures were measured using a ultraviolet-visible-near-IR (UV-VIS-NIR) spectrophotometer (Lambda 950, PerkinElmer, Waltham, MA, USA). The surface morphologies and roughness values of the W-doped samples were measured using a field-emission scanning electron microscope (FE-SEM) (Supra 55, Zeiss, Oberkochen, Germany) and atomic force microscope (Dimension 3100, Veeco, Bruker, Karlsruhe, Germany), respectively. The surface morphologies of damaged sites were observed in detail using an optical microscope (Leica, Solms, Germany) and FE-SEM (Auriga S40, Zeiss, Munich, Germany).

## 3. Results and Discussion

**Figure 3:**
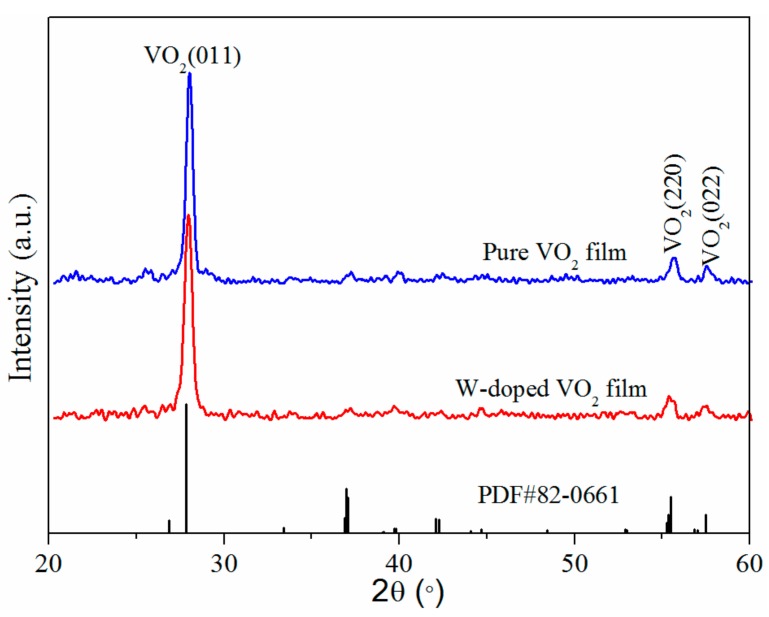
XRD patterns of the pure and W-doped VO_2_ films.

Figure 3 shows XRD patterns of the W-doped and pure VO_2_ thin films, and standard pattern of the VO_2_ phase (PDF#82-0661). The diffraction peaks of the W-doped and pure films are similar and correspond to the characteristic pattern of the VO_2_ phase. It is worth noting that no other impurity peaks can be observed from the XRD patterns, which indicates the formation of single-phase W-doped VO_2_ films. Additionally, the W-doped sample exhibited a preferential growth in the (011) lattice orientation, which is consistent with other reports [23,24]. Moreover, the (011) peak of the W-doped VO_2_ film slightly shifted to a lower 2*θ* value compared with that of the pure VO_2_ film, revealing a small distortion of the lattice as the atomic radii of tungsten and vanadium are different (tungsten introduced in the VO_2_ crystal lattice replaces vanadium).

For a more detailed analysis, the grain size of the film was estimated using Scherrer’s formula, *D* = 0.9*λ*/cos*θ*·Δ*2θ*, where *λ* is the X-ray wavelength, *D* is the crystallite size, *Δ2θ* is the peak full-width at half-maximum (FWHM) (corrected for instrumental broadening), and *θ* is the angle [25]. The most intense peak of (011) was used to calculate the grain sizes, yielding values of 16.2 and 15.5 nm for the W-doped and pure VO_2_ films, respectively. Compared with the pure VO_2_ film, the W-doped VO_2_ film had an excellent crystallinity. These results suggest that the tungsten doping through the proposed vanadium-tungsten alloy target method did not reduce the crystallinity of the W-doped VO_2_ film.

**Figure 4:**
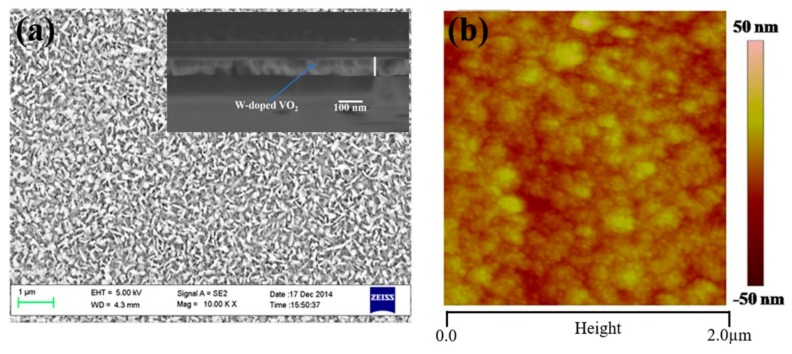
SEM and AFM images of the W-doped VO_2_ film.

Figure 4a shows the surface morphology of the W-doped VO_2_ film. Small rice-like grains were uniformly spread over the film surface, which confirms the uniformity and crystallinity of the W-doped VO_2_ film. The cross-sectional SEM image of the W-doped VO_2_ film in the inset shows the 90-nm-thick W-doped VO_2_. We calculated the transmittance of the film using reported optical constants [26] and thickness of 90 nm, which is consistent with the measured transmittance. A topographic AFM image of the W-doped VO_2_ film is presented in Figure 4b, showing that the surface is continuous and homogeneous. The root-mean-square (RMS) roughness was approximately 6.10 nm, which demonstrates the good surface quality of the W-doped VO_2_ film.

**Figure 5:**
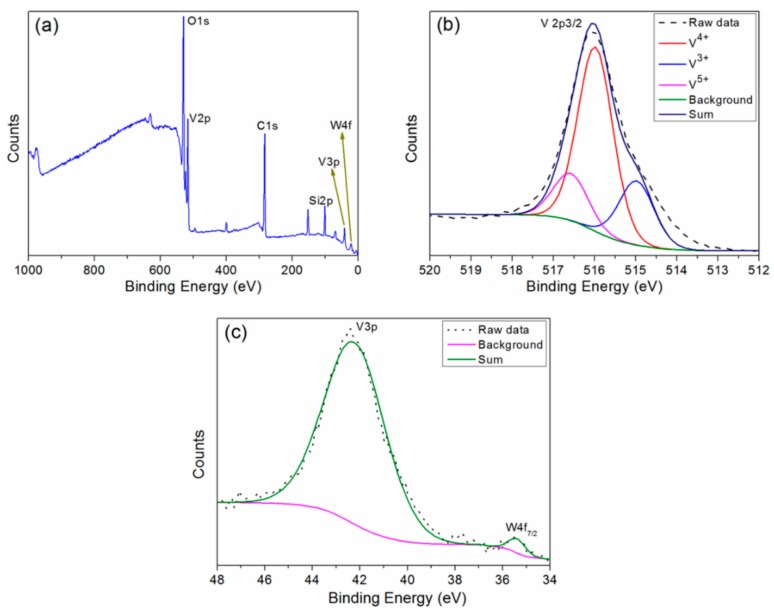
Typical XPS spectra of the W-doped VO_2_ film: (**a**) survey spectrum and high-resolution scans of (**b**) V 2*p*_3/2_ and (**c**) W 4*f*_7/2_.

The chemical state and composition of the tungsten-doped VO_2_ film were investigated by a typical XPS spectrum analysis, as shown in Figure 5. The C 1s peak (284.6 eV) was used for calibration in the XPS spectrum measurements. The obtained wide-range XPS spectrum, shown in Figure 5a, indicates that several elements (V, O, W, Si, and C) were present in the W-doped sample; the presence of carbon could be attributed to surface contamination. Figure 5b presents high-resolution scanning results for the V 2*p*_3/2_ peak, which is the most sensitive peak to the phase changes of VO_2_ [27]. Vanadium was present in three main components, with peaks at 515.0 eV, 516.0 eV, and 516.7 eV corresponding to V^3+^, V^4+^, and V^5+^, respectively [28,29]. The relative concentrations of V^3+^, V^4+^, and V^5+^ in the W-doped VO_2_ film were calculated to be 21.71%, 62.36%, and 15.93%, respectively. This reveals that the W-doped VO_2_ film is good, achieved through the precise control of the oxygen flow rate in the experiment. Additionally, the use of the vanadium–tungsten alloy target to deposit the tungsten-doped VO_2_ film ensured the uniformity of the film and concentration of the doped tungsten, which are beneficial to the formation of the W-doped VO_2_ film. A weak peak of W 4*f*_7/2_ can be observed in Figure 5c, demonstrating the successful doping of the film with tungsten ions. According to the report of Huang et al. [27], the peak of W 4*f*_7/2_ at 35.4 eV corresponds to W^6+^.

**Figure 6:**
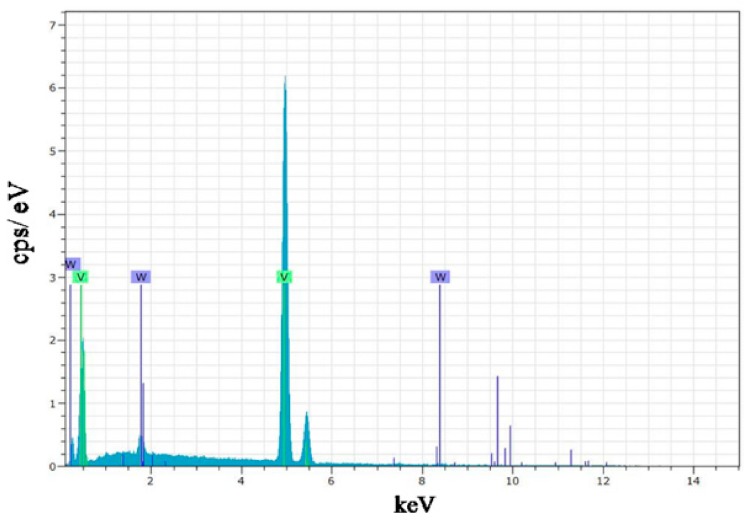
EDS pattern of the W-doped VO_2_ film.

The results of the energy-dispersive spectroscopy (EDS) analysis of the W-doped sample are shown in Figure 6. Peaks attributed to vanadium and tungsten elements can be observed in the EDS pattern, indicating that the VO_2_ film was successfully doped with tungsten. The concentrations of vanadium and tungsten elements were determined to be 99.19 atom% and 0.81 atom%, respectively, in good agreement with the content ratio of the used vanadium–tungsten alloy target. Therefore, the W-doped VO_2_ film had a composition of V_0.9919_W_0.0081_O_2_, consistent with the XPS data.

**Figure 7:**
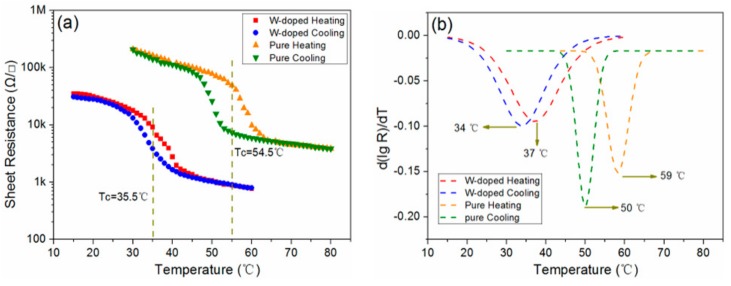
(**a**) Resistance–temperature hysteresis loops and (**b**) phase-transition temperatures of the pure and W-doped VO_2_ films.

The sheet resistances as a function of the temperature for the W-doped and undoped VO_2_ films are shown in Figure 7a. Both samples exhibited the expected thermal hysteresis; however, their sheet resistances were different. The sheet resistance of the W-doped VO_2_ film was lower than that of the pure VO_2_ film. The XPS results in Figure 5 show that the W-doped VO_2_ film contains V_2_O_3_, V_2_O_5_, and VO_2_. However, the pure VO_2_ film mainly consists of VO_2_ and V_2_O_5_, reported in our previous study [30]. The existence of V_2_O_3_ in the film decreases its resistance. For the W-doped VO_2_ film, the addition of tungsten may break V^4+^–V^4+^ bonds, forming V^3+^–W^6+^ and V^3+^–V^4+^ pairs for charge compensation [31]. In this case, the W-doping provides extra electrons to the surrounding V atoms and decreases the resistance in the insulating phase of the W-doped VO_2_ film. Figure 7b shows standard Gaussian-fitted derivative logarithmic plots of the films. The transition temperatures of the W-doped and pure VO_2_ films were 35.5 °C and 54.5 °C, respectively. These results show that the tungsten doping can effectively reduce the transition temperature of VO_2_, consistent with the results of Jin et al. [20], Li et al. [21], and Liang et al. [22], where the tungsten doping linearly decreased the transition temperature of the VO_2_ film at an approximate rate of 23 °C/atom%. The W-doping concentration in this study was approximately 0.81 atom%, determined from the EDS result in Figure 6. Using the above formula, the calculated *T*_c_ for the W-doped VO_2_ film is about 36 °C ((54.5(*T*_c_ of pure VO_2_) − 0.81 × 23) °C ≈ 36 °C), which is close to our experimentally obtained value of 35.5 °C. This demonstrates that the doping concentration of tungsten ions can be precisely controlled using the vanadium–tungsten alloy target.

A W^6+^ ion introduced into the VO_2_ lattice breaks one homopolar V^4+^–V^4+^ bond. For charge compensation, two *d*-shell electrons from the tungsten ion transfer into the neighboring vanadium ions to form V^3+^–W^6+^ along the *a*-axis of the monoclinic VO_2_ cell. With the increase in the fraction of W^6+^ in the lattice, the loss of homopolar V^4+^–V^4+^ bonds progressively increases, leading to destabilization of the semiconductor phase [32,33], which decreases the transition temperature of VO_2_.

**Figure 8:**
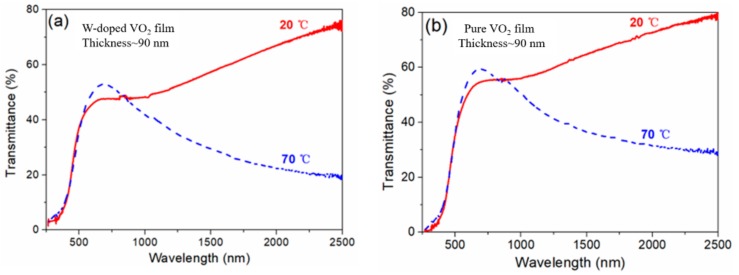
Transmission spectra of the (**a**) W-doped and (**b**) pure VO_2_ films before and after their phase transitions.

The transmittance of the W-doped VO_2_ film was measured in the range of 250 nm to 2500 nm at 20 °C and 70 °C, as shown in Figure 8a. The W-doped sample exhibited a significantly higher IR transmittance in the semiconductive state (20 °C) than that in the metallic state (70 °C), indicating that the VO_2_ film had a high IR-switching performance. The IR transmittance at *λ* = 2500 nm was 75% before the phase transition, which decreased to 19% after the phase transition. The IR modulation value of the W-doped sample at *λ* = 2500 nm reached 56%, better than our previous result for a pure VO_2_ film [34]. In the visible range (390–780 nm), the peak transmittance of the W-doped VO_2_ sample was approximately 50%, which indicates a good luminous transmittance. Moreover, the IR modulation and luminous transmittance of the W-doped VO_2_ film are better than those of reported W-doped VO_2_ films prepared by aerosol-assisted chemical vapor deposition [35] and atmospheric-pressure chemical vapor deposition [36]. Figure 8b shows the optical transmittance of the pure VO_2_ film with a thickness (90 nm) equal to that of the W-doped VO_2_ film. The luminous and IR optical transmittances of the W-doped VO_2_ film are slightly lower than those of the pure VO_2_ film.

**Figure 9:**
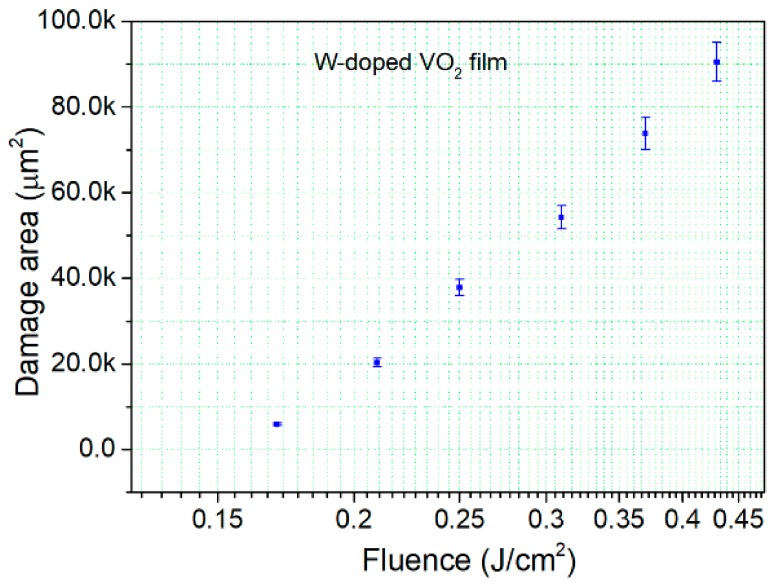
Damage area as a function of the fluence for the W-doped VO_2_ film; the error bars were obtained using the practically measured energy fluence.

**Figure 10:**
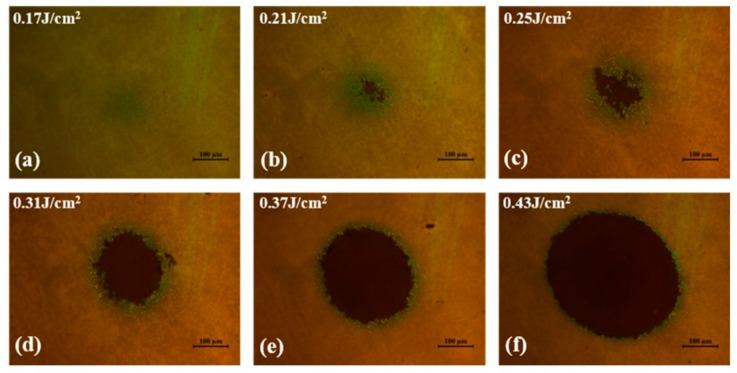
Optical morphologies of the W-doped VO_2_ film under different laser fluence levels.

Figure 9 shows the statistical results of the laser damage test of the W-doped VO_2_ film. The one-on-one LIDT of the W-doped VO_2_ film was 0.14 ± 0.01 J/cm^2^, estimated by the damaged-area-extrapolation method, using the relationship between the logarithm of the laser fluence and damaged area [37,38]. The optical morphologies of portions of the W-doped VO_2_ film damaged by the fs-laser are shown in Figure 10. The damage mechanism depends on the fluence level. At a fluence level just above the damage threshold, the damaged site is characterized by surface coloring, which indicates an enhanced roughness. The size of the colored region increases with the incident fluence; ablation in the center of the damaged site occurs when the fluence reaches 0.25 J/cm^2^. At higher fluence levels, the ablated region develops significantly faster than the colored region and eventually occupies most of the damaged site.

**Figure 11:**
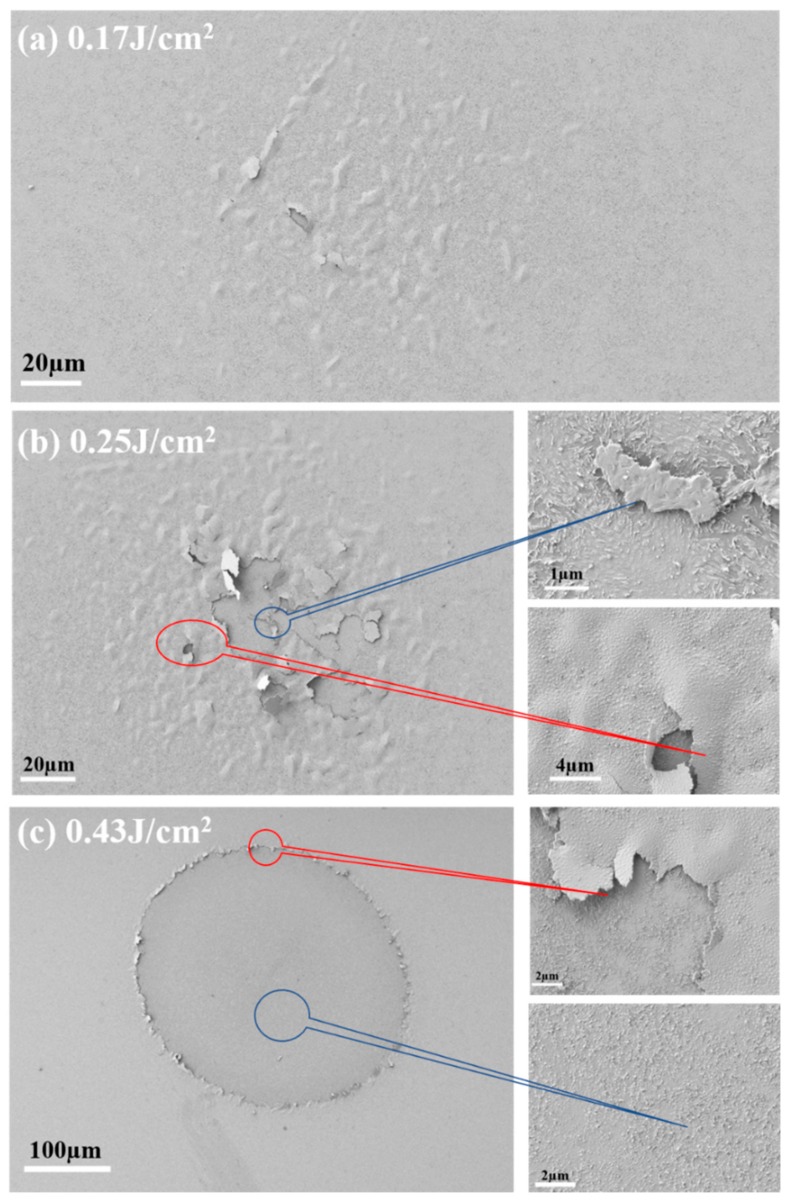
SEM morphologies of the W-doped VO_2_ film under different fluence levels of (**a**) 0.17, (**b**) 0.25, and (**c**) 0.43 J/cm^2^. The right-hand subfigures in (**b**,**c**) are partial enlarged images.

The high-resolution SEM images in Figure 11 reveal that the colored region contained abundant isolated microbuckling nodules; certain microbuckling nodules were broken owing to higher localized energies. The isolated microbuckling nodules can lead to fluctuations of the coating surface and roughness enhancement. With the increase in the fluence level, more microbuckling nodules were broken. The ablation in the center of the damaged site was invisible until the broken microbuckling nodules joined. Therefore, there are two damage thresholds: buckling and breaking thresholds. The former is related to the surface coloring phenomenon observed in the optical morphology of the film, while the latter is related to the ablation phenomenon. A transitive threshold (~0.21 J/cm^2^) was defined to determine the fluence required to transition from isolated microbuckling nodules to integral ablation. The joining process of the isolated microbuckling nodules can be observed from the edge of the damaged site in Figure 11c. Similar phenomena have been reported in our previous study for dielectric coatings damaged using an 800-fs laser [39]. Large amounts of defects or impurities may exist at the surface of the W-doped VO_2_ film due to the metal doping. At a fluence level just above the damage threshold, coating defects can induce local light field enhancements at the nanoscale. Additionally, the absorption coefficient of defects is high [37]. The above two factors may contribute to the increase in density of electrons surrounding defects during the pulse irradiation. When the free-electron density reached a critical plasma density, the laser energy was strongly absorbed by the forming plasma, leading to small damage spots [40]. This phenomenon can be observed in Figure 10a. With the increase in the pulse energy, the outline of the damaged area becomes clearer and large-scale ablation can be observed in Figure 10b–f. In this case, the damage in the fs-regime is intrinsic and the coating spalling becomes more significant than the impact of defects. The role of defects in laser-induced modifications of dielectric materials has also been studied by Laurence et al. [41] and Gallais et al. [42]. According to the results of this study, the defect-related damage induced by fs-lasers is important for semiconductive materials. It is necessary to study the damaging process in detail, which can be a subject of a following study.

## 4. Conclusions

Thermochromic tungsten-doped VO_2_ thin films were successfully fabricated using a W–V alloy target. The damage behavior of the W-doped VO_2_ films under a fs-laser irradiation was investigated experimentally and theoretically. The results showed that 0.81 atom% W^6+^ replaced vanadium in the lattice of the W-doped sample. The metal-insulator transition temperature of the W-doped VO_2_ film was approximately 35.5 °C, which is near room temperature. The W-doped VO_2_ film exhibited an excellent thermochromic performance. Laser-induced damage tests were performed with 800-nm 60-fs pulses; the LIDT for the W-doped VO_2_ film was approximately 0.14 J/cm^2^. The defect-related damage induced by fs-lasers is relevant for W-doped VO_2_ films. This study provided valuable insights regarding VO_2_ films used for laser protection.
